# Production Systems and Feeding Strategies in the Aromatic Fingerprinting of Animal-Derived Foods: Invited Review

**DOI:** 10.3390/foods14193400

**Published:** 2025-10-01

**Authors:** Eric N. Ponnampalam, Gauri Jairath, Ishaya U. Gadzama, Long Li, Sarusha Santhiravel, Chunhui Ma, Mónica Flores, Hasitha Priyashantha

**Affiliations:** 1 School of Agriculture, Food and Ecosystem Sciences, Faculty of Science, The University of Melbourne, Parkville, VIC 3010, Australia; 2 Department of Livestock Products Technology, ICAR-Indian Veterinary Research Institute, Regional Station, Palampur 176061, Himachal Pradesh, India; 3 School of Agriculture and Food Sustainability, University of Queensland, Gatton, QLD 4343, Australia; ishaya.gadzama@adelaide.edu.au; 4 College of Animal Science and Technology, Shihezi University, Shihezi 832000, China; ll18831851783@163.com (L.L.); chunhuiima@163.com (C.M.); 5 Department of Biochemistry, Memorial University of Newfoundland, St. John’s, NL A1C 5S7, Canada; ssanthiravel@mun.ca; 6 Department of Food Science, Instituto de Agroquímica y Tecnología de Alimentos (CSIC), 46980 Paterna, Spain; mflores@iata.csic.es; 7 Department of Molecular Sciences, Swedish University of Agricultural Sciences, Box 7015, SE-750 07 Uppsala, Sweden; hasithap@agri.ruh.ac.lk; 8 Department of Animal Science, Faculty of Agriculture, University of Ruhuna, Mapalana, Kamburupitiya 81100, Sri Lanka

**Keywords:** volatile organic compounds, animal feeds, meat and milk production, farming practices, consumer trust

## Abstract

Aroma and flavor are central to consumer perception, product acceptance, and market positioning of animal-derived foods such as meat, milk, and eggs. These sensory traits arise from volatile organic compounds (VOCs) formed via lipid oxidation (e.g., hexanal, nonanal), Maillard/Strecker chemistry (e.g., pyrazines, furans), thiamine degradation (e.g., 2-methyl-3-furanthiol, thiazoles), and microbial metabolism, and are modulated by species, diet, husbandry, and post-harvest processing. Despite extensive research on food volatiles, there is still no unified framework spanning meat, milk, and eggs that connects production factors with VOC pathways and links them to sensory traits and consumer behavior. This review explores how production systems, feeding strategies, and processing shape VOC profiles, creating distinct aroma “fingerprints” in meat, milk, and eggs, and assesses their value as markers of quality, authenticity, and traceability. We have also summarized the advances in analytical techniques for aroma fingerprinting, with emphasis on GC–MS, GC–IMS, and electronic-nose approaches, and discuss links between key VOCs and sensory patterns (e.g., grassy, nutty, buttery, rancid) that influence consumer perception and willingness-to-pay. These patterns reflect differences in production and processing and can support regulatory claims, provenance verification, and label integrity. In practice, such markers can help producers tailor feeding and processing for flavor outcomes, assist regulators in verifying claims such as “organic” or “free-range,” and enable consumers to make informed choices. Integrating VOC profiling with production data and chemometric/machine learning pipelines can enable robust traceability tools and sensory-driven product differentiation, supporting transparent, value-added livestock products. Thus, this review integrates production variables, biochemical pathways, and analytical platforms to outline a research agenda toward standardized, transferable VOC-based tools for authentication and label integrity.

## 1. Introduction

In recent years, the sensory quality of animal-derived foods including milk, meat, and eggs has gained increasing importance, not only as a marker of consumer preference but also as a proxy for authenticity and production transparency. Among various sensory attributes, aroma and flavor stand out as key determinants of food acceptability, directly influencing purchasing decisions and perceived value [1]. These characteristics are largely governed by volatile organic compounds (VOCs), which are generated by thermal degradation of flavor precursors that originate during both on-farm (e.g., metabolism, microbial interactions) and post-farm (e.g., processing/cooking and storage) stages [2]. These volatiles act as chemical fingerprints, offering insights into production systems, animal diets, and environmental exposures [3].

Advanced analytical technologies such as headspace–gas chromatography–mass spectrometry–olfactometry (HS–GC–MS–O), gas chromatography–ion mobility spectrometry (GC–IMS), and electronic nose (E-nose) systems have been instrumental in decoding these complex aroma profiles. GC–MS remains the gold standard for separating and identifying hundreds of volatile compounds with high specificity, while GC–IMS offers rapid analysis of complex mixtures with portable configurations suitable for routine food authentication. E-nose devices, which rely on chemical sensor arrays to generate holistic “smell prints,” provide fast and non-destructive profiling, making them valuable for quality assurance at industrial scale. Alongside these, emerging spectroscopic techniques such as Fourier-transform infrared (FTIR), near-infrared (NIR), and Raman spectroscopy provide rapid, non-invasive fingerprinting of VOC mixtures. Together, these methods capture the breadth of aroma-active molecules, ranging from aldehydes such as hexanal (lipid oxidation marker) and Strecker-derived methional, to fermentation products like 2,3-butanedione and thermally derived heterocycles such as pyrazines and furans, each of which contributes to distinct sensory signatures (“fruity,” “mushroom,” “fatty,” and “scorch” notes in water-boiled salted duck) [4,5].

Several intrinsic and extrinsic factors further influence the VOC composition of animal products. These include species, breed, age, sex, diet, production environment, and microbial communities. Among these, animal diet exerts a particularly significant influence, with pasture-based systems consistently associated with richer aroma profiles and healthier lipid content, such as higher levels of alpha-linolenic acid and other omega-3 fatty acids [6]. These bioactive lipids not only contribute to the nutritional profile of the meat or milk but also serve as precursors to flavor-active compounds developed during cooking through the Maillard reaction and lipid oxidation [7]. For example, heat treatment markedly alters the VOCs and sensory attributes of milk, with 65 °C for 30 min preserving a flavor profile close to raw milk, while 135 °C treatment leads to distinct bitterness and the formation of characteristic VOCs such as furfural, 2-heptanone, and 4,7-dimethyl-undecane [8]. These findings show how processing temperatures influence milk flavor chemistry and provide useful insights for optimizing quality control in dairy production.

Furthermore, microbial activity plays a vital role in VOC formation. Biçer et al. [9] reported breed-specific differences in milk microbiota and VOC profiles among Merino, Lacaune, and Assaf sheep, with distinct correlations between microbial genera (e.g., *Lactobacillus*, *Salinicoccus*, *Psychrobacter*) and volatiles such as ketones and alkanes. These findings emphasize the interdependence of animal genetics, microbial ecology, and sensory traits.

Beyond biochemical drivers, consumer perception is increasingly influenced by contextual and ethical cues, including labels such as “organic” and “pasture-raised.” Products from grazing systems are often associated with enhanced flavor, nutritional benefits, and environmental sustainability [10]. However, despite these positive associations, verifying such claims remains complex due to variability in production practices and overlap in sensory traits between conventional and alternative systems [11]. The presence of plant secondary metabolites in animal diets—such as polyphenols, flavonoids, carotenoids, and essential oils—can further influence aroma and shelf-life along with prevention of rancidity, adding functional value to the product [12,13]. Traceability standards such as ISO 22005:2007 and EU Regulation (EC) No. 178/2002 also provide a legal and operational framework for verifying the origin and safety of animal-derived foods, and quality assurance seals such as Protected Designation of Origin (PDO), Protected Geographical Indication (PGI), Organic, Halal, and Kosher certifications extend these frameworks as market signals that communicate authenticity and adherence to production standards; collectively, they connect well with VOC profiling, since aroma fingerprints can serve as analytical markers that reinforce such labeling claims.

**Figure 1:**
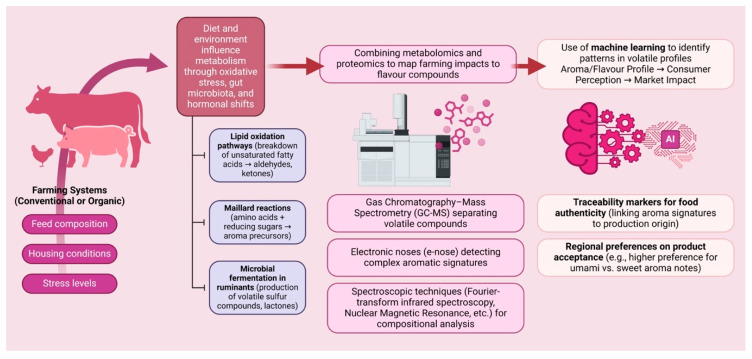
Interconnected pathways linking farming systems, metabolic processes, and VOC formation in animal-derived foods.

At the same time, consumer willingness to pay premiums for products perceived as natural, ethical, or health-promoting highlights the growing need for traceability and authenticity verification based on scientific evidence [14]. These interconnections are illustrated in Figure 1, which outlines the conceptual framework linking production systems, metabolic pathways, and VOC fingerprinting. Despite these developments, a considerable gap remains in linking biochemical mechanisms of aroma development with agricultural practices and consumer behavior in a unified framework.

Therefore, this review aims to present a comprehensive synthesis of the factors driving aromatic fingerprinting in animal-derived foods, examining how production systems, animal diets, microbial communities, heat treatment and environmental conditions interact to shape VOC profiles via generation of diverse VOCs precursors. It further explores the emerging analytical methods used to detect these compounds and discusses how such sensory signatures influence quality assessment, consumer perception, and food authentication. However, this review does not cover added-ingredient formulations of milk, meat, or eggs (e.g., yogurt, burger patties) or the VOC effects arising from interactions with such ingredients during processing.

## 2. Methodological Approach

This review was drafted using a structured literature search across scientific databases including Scopus, Web of Science, PubMed, and ScienceDirect. Keywords utilized included “*volatile organic compounds*,” “*meat aroma*,” “*livestock aroma*,” “*farming systems*,” “*pasture-fed*,” “*grain-fed*,” “*organic livestock*,” “*GC–MS*,” “*GC–IMS*,” “*E-nose*,” “*FTIR*,” and “*food authenticity,*” focusing largely on studies published in a decade from 2015 to 2025. Although Ni et al. [3] identified 611 meat-specific VOC studies from 2000 to 2020, our broader thematic scope to include dairy, eggs, and authenticity justified a broader search. The initial query retrieved 2175 records; 620 duplicates were removed. Following title/abstract screening of 1555 records (excluding 1175 irrelevant studies, e.g., plant-based VOCs, sensory-only reports), 380 full-texts were assessed. About 203 were excluded for reasons ranging from lack of VOC analysis to insufficient methods or irrelevant matrices. Ultimately, 177 primary studies were included in the synthesis, aligning with the reference list. Articles were screened based on relevance, prioritizing peer-reviewed research investigating the biochemical mechanisms underlying aroma and flavor development in animal-derived products (meat, dairy and eggs) under varied rearing conditions.

Selected studies were further synthesized thematically to explore the following:

- The effects of conventional, organic, and sustainable farming systems on animal metabolism and the generation of aroma precursors.
- The interaction between animal breeds and environmental conditions influencing aromatic profiles.
- The application of advanced analytical technologies (e.g., GC–MS, GC–IMS, electronic nose, FTIR spectroscopy) in VOC profiling and fingerprinting.

Information was qualitatively synthesized, emphasizing key biochemical pathways such as lipid oxidation, Maillard reactions, microbial metabolism, and thiamine degradation. Quantitative data, including odor activity values (OAVs) and VOC concentrations, were integrated when available to support comparative analyses. This methodological approach facilitated the identification of specific VOCs as biomarkers associated with different farming practices, thereby linking production methods to product quality, authenticity, traceability, and consumer perception.

## 3. Volatile Organic Compounds: Sources and Biogenesis

**Figure 2:**
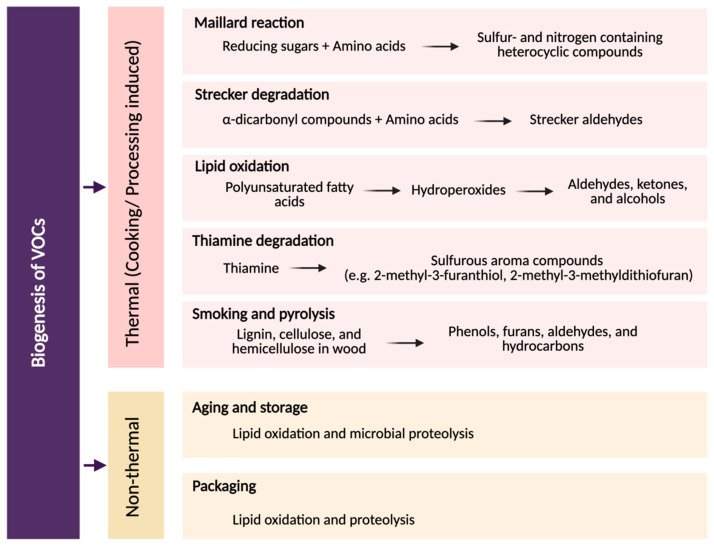
Different ways of VOC formation in foods.

VOCs are low-molecular-weight molecules that play a pivotal role in defining the aroma, flavor, and overall sensory quality of animal-derived foods including meat, milk and eggs by providing species-specific aromatic signatures, indicating freshness or spoilage, and influencing consumer acceptability and quality assurance [15]. These compounds are generated through various biochemical pathways, taking place during thermal treatments including lipid oxidation, and Maillard reactions, or by the microbial metabolism present in the raw materials. Among all, Maillard and Strecker reactions generate a wide range of volatiles that significantly contribute to roasted, nutty, and meaty aromas. Representative compounds include aldehydes such as 2-methylbutanal and 3-methylbutanal, pyrazines (e.g., 2,5-dimethylpyrazine, 2,6-dimethylpyrazine), furans such as furfural and 2-acetylfuran, and sulfur-containing molecules like 2-methyl-3-furanthiol and thiazoles. Understanding how and why particular VOCs are generated is key to interpreting aroma fingerprints. The biogenesis of VOCs in foods can be broadly categorized into non-thermal (enzymatic, oxidative, and microbial) processes and thermal (cooking/processing-induced) processes as shown in Figure 2 [16]. To explain further, in this section below we will discuss the formation of VOCs in food products, governed by both thermal and non-thermal biochemical and physicochemical reactions. These reactions act on endogenous precursors such as amino acids, sugars, free fatty acids, vitamins, and nucleotides, generating a diverse array of aroma-active molecules that define the sensory quality and identity of animal-derived products.

### 3.1. Thermal Pathways

Thermal processing methods—such as roasting, grilling, frying, boiling, and smoking—are primary drivers of VOC formation in food products, especially meat. Major thermally driven pathways include the Maillard reaction, Strecker degradation, lipid oxidation, and thiamine degradation.

#### 3.1.1. Maillard Reaction

**Figure 3:**
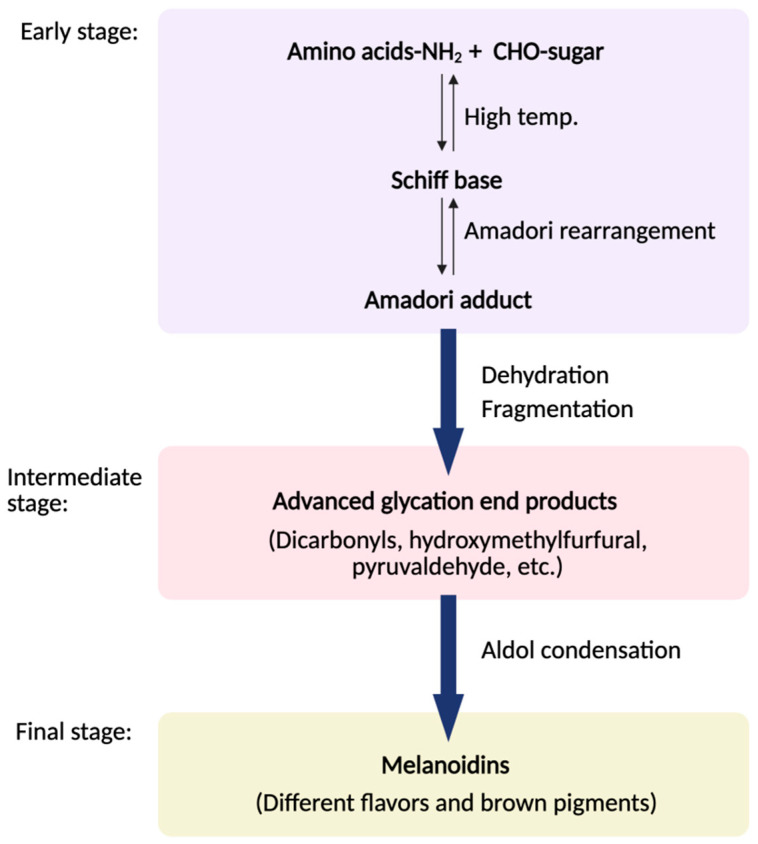
Stages of Maillard reaction and flavor formation.

Maillard reaction can be defined as a non-enzymatic browning reaction occurring between reducing sugar and amino acid, primarily during heating of food. In general, meat is prepared at high temperatures through methods such as frying, roasting, boiling, or baking in an oven, during which it undergoes the Maillard reaction [17]. It unfolds in three stages (Figure 3): sugar-amino acid condensation and Amadori rearrangement to form 1-amino-1-deoxy-2-ketose; intermediate dehydration and fragmentation into hydroxymethylfurfural, pyruvaldehyde, and diacetyl alongside amino acid degradation; and aldol condensation to heterocyclic melanoidins [18]. Under low-moisture, high-heat conditions, these pathways generate volatile heterocycles—pyrazines, thiazoles, thiophenes, and furans—that impart roasted, nutty, and meaty aromas [19]. In grilled beef and lamb, 2,5-dimethylpyrazine and trimethylpyrazine increase markedly with heat and, thanks to their low odor thresholds (~1.75 µg/kg and ~350 µg/kg), exert pronounced sensory effects [20]. In dairy, thermal treatments such as pasteurization, UHT, spray drying, and baking drive Maillard reactions between lactose and the free amino groups of casein and whey, producing ketones, aldehydes, furans, pyrazines, and sulfur compounds, with the VOC profile determined by reactant types and processing conditions [21]. However, it is also important to note that heat treatment can generate undesirable compounds such as acrylamide, which has raised concern due to its potential toxicity and carcinogenicity in thermally processed foods [20].

#### 3.1.2. Strecker Degradation

It is a key pathway linked to the Maillard reaction, in which amino acids react with α-dicarbonyl compounds (e.g., deoxyosones) to yield Strecker aldehydes—important volatiles in cooked meat aroma. This reaction leads to the formation of aldehydes with one carbon less than their corresponding amino acid, such as 3-methylbutanal from leucine, phenylacetaldehyde from phenylalanine, and methional from methionine. These compounds are associated with “malty”, “chocolate-like”, “sweet”, or “cooked-potato aromas, respectively [4,22]. For example, methional, imparting a characteristic potato”-like note, has been consistently reported as a key Strecker aldehyde in cooked meats. 3-methylbutanal, often described as malty or cocoa-like, is frequently detected in grilled and roasted pork and beef [16]. These aldehydes may also act as precursors to nitrogen-containing heterocycles such as pyrazines when reacting with aminoketones under heat, further contributing to roasted and nutty flavors in thermally processed meats [22].

#### 3.1.3. Lipid Oxidation

Lipid oxidation is a major source of aroma-active VOCs in meat during cooking and storage. Polyunsaturated fatty acids (linoleic, arachidonic) form hydroperoxides that cleave into aldehydes, ketones and alcohol, most notably hexanal, a low-threshold marker of warmed-over flavor with a grassy-fatty note [22]. Heating also degrades phospholipids and triglycerides, releasing short-chain fatty acids that subsequently oxidize into secondary volatiles. Although lipid-derived VOCs generally require higher concentrations for detection than Maillard heterocycles [23], saturated and unsaturated C_6_–C_10_ aldehydes remain dominant in all cooked-meat profiles [24]. Other key lipid oxidation products include 1-octen-3-ol, which lends a mushroom aroma [4], and 2-heptanone, which adds subtle sweet-fruity notes [25]. Certain unsaturated aldehydes—(E)-2-heptenal and (E,E)-2,4-decadienal—interact with Maillard intermediates, inhibiting Amadori rearrangements and suppressing the formation of sulfur-heterocycles (e.g., furanthiols, thiophenes) [23,25]. These cross-reactions exemplify the tight linkage between lipid oxidation and Maillard chemistry, in which lipid-derived carbonyls can either divert precursors away from heterocyclic sulfur compounds or generate Strecker-type aldehydes that modify flavor balance. Consequently, the accumulation of lipid oxidation products not only contributes directly to fatty and rancid notes but also indirectly reshapes the profile of meaty, roasted aromas that are otherwise dominated by thiamine degradation and sulfur amino acid pathways [26]. This shifts cooked-meat aroma toward greener or slightly rancid notes in polyunsaturated-rich cuts [23,25]. By generating both characteristic green-fatty aromas and modulating Maillard-derived flavors, lipid oxidation plays a dual and indispensable role in shaping meat’s sensory character.

#### 3.1.4. Thiamine Degradation

Thiamine (vitamin B1), a sulfur- and nitrogen-containing vitamin, is a significant precursor of sulfurous aroma compounds formed during cooking. Its content in animal foods ranges from very low in milk (0.03–0.06 mg/100 g) to moderate in eggs (0.09–0.30 mg/100 g) and higher in meats, spanning beef (0.05–0.15 mg/100 g) and chicken (0.04–0.11 mg/100 g) to pork (0.5–1.16 mg/100 g), lamb organs (0.38–0.51 mg/100 g), and chicken liver (0.61 mg/100 g) [27]. In this section, thiamine degradation in pork derived products have been discussed owing to its higher thiamine content. Its thermal degradation produces highly odor-active volatiles such as 2-methyl-3-furanthiol, 2-methyl-3-methyldithiofuran, and bis (2-methyl-3-furyl) disulfide, all of which contribute to the meaty, boiled, and roasted aroma of pork and cooked ham. These compounds have exceptionally low odor thresholds and are considered among the most potent meat odorants [28]. The thermal degradation of thiamine is a complex process involving multiple reaction pathways that generate a variety of meat-like flavor compounds. The complexity of this reaction results in the formation of a wide range of volatile compounds that contribute to the distinctive meat-like flavor [29]. The degradation pathways involve intermediates such as 5-hydroxy-3-mercapto-2-pentanone, with product formation depending on factors like pH, temperature, and phosphate availability [16]. Studies cited in the same review show that thiamine plays a more critical role in pork flavor than in beef or chicken, as the addition of thiamine increased meaty aroma only in pork [30]. Furthermore, these thiamine-derived volatiles are found not only in heat-treated pork but also in dry-fermented products, suggesting alternative non-thermal routes such as microbial or Maillard interactions [31].

### 3.2. Non-Thermal Pathways

#### 3.2.1. Microbial Metabolism

Microbial metabolism also generates distinctive VOCs in animal-derived foods, particularly during fermentation, curing, or storage. For instance, in fermented sausages, *Lactobacillus* spp. and *Staphylococcus* spp. produce aldehydes such as 3-methylbutanal and 2-methylbutanal, with reported concentrations of 50–200 µg/kg and odor activity values (OAVs) > 10, indicating strong sensory relevance [16]. In blue-veined cheeses, *Penicillium roqueforti* forms methyl ketones such as 2-heptanone and 2-nonanone at concentrations above 1 mg/kg, with OAVs exceeding threshold values, imparting sharp, piquant notes [16]. Similarly, *Brochothrix thermosphacta* contributes spoilage markers like ethyl acetate (1–3 mg/kg) and 3-methylbutanal, both of which have high OAVs and correlate with sour or off-flavors [23].

#### 3.2.2. Aging and Storage

During aging and storage of meat, endogenous muscle enzymes and microbial enzymes can breakdown protein (proteolysis) and lipids (lipolysis). The breakdown products such as peptides, amino acids, and free fatty acids can undergo further breakdown generating VOCs in wet and dry-aged beef [32]. Dry aging substantially alters the volatile profile of beef, primarily via lipid oxidation and microbial proteolysis, leading to higher levels of Strecker aldehydes (2-methylbutanal, 2-methylpropanal) and sulfur compounds (e.g., 2-methyl-2-propanethiol), which are known to impart roasted or nutty notes to beef flavor. In contrast, compounds such as propanal and trimethylamine, also elevated during dry aging, are markers of lipid oxidation and microbial activity, respectively, and may contribute fewer desirable notes [32]. Propanal, a marker of lipid oxidation, increases significantly during dry aging due to oxygen exposure, whereas its formation is largely suppressed in vacuum-packed wet aging [32,33]. Strecker aldehydes such as 2-methylbutanal and 2-methylpropanal arise from isoleucine and valine degradation, primarily released through endogenous muscle proteases during post-mortem aging, with additional contributions from microbial enzymes at the meat surface in later stages of dry aging [32]. The late-phase spike in trimethylamine and 1-butanamine further supports enhanced microbial enzyme activity on proteins [34]. Additionally, esters like ethyl propanoate increased via microbial esterification while 2-methylpropanoic acid, detected only in dry-aged samples post-day 14, suggests oxidation of Strecker aldehydes [35]. Moreover, lactic acid bacteria ferment residual sugars into lactic acid, acetic acid, ethanol, carbon dioxide, and various esters.

#### 3.2.3. Packaging

The development of aroma and flavors in meat due to packaging is a complex process influenced by the type of packaging material, storage conditions, and duration. Packaging affects meat flavor both directly (e.g., by transferring odors or restricting gas exchange) and indirectly (e.g., by altering microbial activity or oxidation processes). Vacuum packaging reduces contact with oxygen, thereby limiting oxidation, while modified atmosphere packaging alters the levels of oxygen, nitrogen, and carbon dioxide to control oxidation and microbial growth. Moreover, active packaging includes oxygen scavengers and antimicrobials to help preserve aroma. Aroma-imparting films coated with natural flavorings (e.g., essential oils) are an emerging technique used to introduce herbal or smoky notes.

In a comparative study, Bhadury et al. [36] identified 35 VOCs from beef stored under modified atmosphere packaging (MAP), vacuum packaging (VP), and cling-wrapped packaging (CP packaging), using solid-phase microextraction GC-accurate mass spectrometry (SPME-GC–accQTOFMS). Only three compounds carbon disulphide (CS_2_), acetoin, and 2-vinyloxyethanol—were common across all systems, indicating strong packaging-dependent variation. Emerging technologies such as pulsed electric fields, cold plasma, ultrasound, and high-pressure processing also alter VOC generation. For example, cold plasma can enhance lipid oxidation, increasing levels of aldehydes and ketones, while ultrasound improves proteolysis and enhances the release of volatile precursors [16].

## 4. Farming Practices and Aromatic Profiling of Animal-Derived Food

Production variables including animal age, breed, diet and rearing system collectively determine fatty acid composition in animal-derived products, which in turn govern VOC profiles in them during storage and cooking. Meat lipids differ across species, for instance, ruminant meats (beef, lamb) are typically dominated by saturated fatty acids (SFA; C16:0, C18:0) and lower PUFA due to ruminal biohydrogenation, whereas non-ruminant meats such as pork and poultry contain more unsaturated fatty acids, particularly oleic acid (C18:1n-9) and linoleic acid (C18:2n-6) [37,38]. Milk fat contains ~65–70% SFA, including short- and medium-chain fatty acids (C4:0–C12:0), which contribute uniquely to its nutritional and sensory properties [39], while eggs are enriched in long-chain PUFA, notably linoleic acid (C18:2n-6) and arachidonic acid (C20:4n-6), with n-3 PUFA (ALA, EPA, DHA) levels being highly diet-dependent [40]. These compositional differences set the stage for aroma development older animals with higher intramuscular fat yield more lipid-derived volatiles such as hexanal and 1-octen-3-ol [41], and breed influences egg VOC balance, with White Leghorn and Hy-Line Brown eggs containing ~80% aldehydes compared to Jing Fen eggs [42]. Diet further drives species-specific signatures: pasture feeding enriches ruminant meat and milk with terpenes and branched-chain fatty acids (BCFA), imparting grassy and dairy-like notes, while grain feeding elevates n-6 PUFA and oleic acid, favoring Maillard-derived aldehydes such as nonanal that contribute to roasted flavors [25]. In dairy, pasture versus silage diets alter polyphenol, sulfur, ketone and free fatty acid contents [43]. Free-range and organic systems introduce further VOC diversity through varied foraging and environmental exposures. Processing techniques—dry aging, sous-vide cooking and fermentation—generate signature compounds (methional, furans, sulfur volatiles) and influence oxidative stability; dietary antioxidants like vitamin E can mitigate off-flavors [44,45].

### 4.1. VOCs in Milk

VOCs are among the most important contributors to the flavor and sensory appeal of milk and dairy products. They originate from lipolysis, proteolysis, and microbial fermentation during storage, processing, and cheese ripening, and include a wide range of aldehydes, ketones, esters, and sulfur compounds [9]. Because their profiles vary with breed, diet, stage of lactation, and processing conditions, VOCs serve not only as quality markers but also as potential tools for product differentiation. Feeding systems have a particularly strong influence on milk VOCs (Table 1). Pasture feeding typically increases phenolic and sulfur-derived compounds, while hay-based diets lead to higher levels of free fatty acids and lactones [43]. Total mixed ration (TMR) systems show a different trend, with cheeses produced under TMR containing more alcohols and esters but comparatively less acetic acid than those from animals managed under separate feeding regimes [46]. Breed-related effects are also well documented. For instance, VOCs in Merino sheep milk are dominated by ketones (about 72% of the total), whereas Lacaune and Assaf milks contain higher proportions of hydrocarbons [9]. Clear species-specific differences further enrich the VOC composition of dairy products. More than 70 aroma-active compounds have been identified in bovine milk, many of which are nitrogen heterocycles and oxidation products of linolenic acid that contribute significantly to flavor development [47]. Buffalo milk is distinguished by unique odorants such as 1-octen-3-one and indole, giving it a sensory profile that differs noticeably from cow, sheep, and goat milk [48]. Understanding these variations enables producers to tailor feeding and processing to enhance desirable aromas and suppress off-flavors, while supporting authenticity verification and premium marketing based on VOC fingerprints.

### 4.2. VOCs in Muscle Foods

VOCs are central to the aroma and flavor characteristics of muscle foods and play a crucial role in defining sensory quality, consumer acceptance, and market value. Generated through lipid oxidation, Maillard reactions, thiamine degradation, and microbial activity, VOC reflect a dynamic interplay between biological, dietary, and environmental factors. Their profiles are influenced by species-specific metabolic processes as well as by production practices, including breed, feeding system, and postmortem handling. This section explores the formation and modulation of VOCs in beef, lamb, poultry and rabbit drawing from recent advances in the understanding of how these compounds contribute to food quality and authenticity.

#### 4.2.1. VOCs in Beef

Beef aroma arises from lipid oxidation (aldehydes like hexanal) and Maillard reactions (sulfur heterocycles) that develop during cooking and aging [15]. For instance, Francisco et al. [53] showed that Canchim steers (5/8 Charolais × 3/8 Zebu) fed a pellet diet of peanut shell, corn and soybean meal and then dry-aged for 28 days produced increased methional (cheddar-like) and furan (roasted-beef) volatiles, which coincided with enhanced tenderness and a clearly preferred flavor profile. Similarly, supplementing crossbred steers with benzoic acid (0.5% DM for 98 days) boosted beefy and roasted notes without altering shear force, texture or oxidative stability [54]. More generally, finishing on high-energy or grain-based rations enhances Maillard-derived roasted and umami notes [55], as exemplified by grain-fed beef’s elevated nonanal and stronger “beefy” flavor [25]. In contrast, grass-fed beef shows elevated hexanal and various terpenoids, conferring grassy or gamey aromas along with improved oxidative stability but slightly reduced tenderness [25,55]. The effect of breed and diet on beef quality has been summarized in Table 2.

#### 4.2.2. VOCs in Sheep and Lamb Meat

Sheep and lamb meat VOCs—notably branched-chain fatty acids (BCFAs), terpenes and aldehydes—are central to their characteristic aroma and consumer appeal [60,61]. As detailed in Table 3, feeding regime markedly alters these profiles: pasture-fed lambs accumulate higher terpenes and BCFAs—especially 4-methyloctanoic and 4-ethyloctanoic acids—that underpin the classic “mutton” flavor, whereas grain finishing boosts Maillard-derived roasted volatiles [62]. Concentrate-based diets further enhance overall meat aroma compared with silage systems, which can impart off-flavors [35]. These findings enable producers to fine-tune diet and management strategies to amplify desirable aromas or suppress off-notes, thereby supporting product differentiation and premium marketing through authentic flavor signatures.

#### 4.2.3. VOCs in Poultry Meat

Poultry products exhibit VOC profiles dominated by lipid-oxidation and Maillard-reaction derivatives—primarily aldehydes (hexanal, nonanal), sulfur volatiles (2-methyl-3-furanthiol) and ketones [25]. Dietary strategies, age of broiler and environment reshape these profiles as detailed in Table 4. For instance, black cumin seed meal (20–60 g/kg) elevates pyrazines and aldehydes while improving texture and water-holding capacity [69]; Sacha inchi oil (0.5%) boosts ω-3 fatty acids and associated VOCs but increases oxidative susceptibility [45]; and housefly larva meal (5%) introduces sulfurous thiols without sensory drawbacks [70]. Bird age influences complexity too—150-day-old Daheng broilers yield higher hexanal and 1-octen-3-ol [41]. Processing further modulates VOCs: fermented coffee pericarp (2.5%) raises aldehydes and ketones while reducing drip loss by 12% [71] and roasting generates more aldehydes than boiling or sous-vide [72]. Consequently, tailoring VOCs via diet and processing can enhance poultry flavor and shelf life—delivering consistent quality and ω-3 enrichment—while still requiring strategies to mitigate oxidative instability when using polyunsaturated fats [45].

Thus, diet and processing shift poultry VOCs in ways consumers immediately perceive: unsaturated fat feeding raises aldehydes like hexanal (fresh at low, rancid at high), antioxidants suppress off-notes while boosting pleasant mushroom-like alcohols, and cooking generates desirable roasted pyrazines but excessive heat or storage drives sulfur volatiles and spoilage amines that undermine freshness.

#### 4.2.4. VOCs in Rabbit Meat

Rabbit meat, characterized by high PUFA content and low total fat, is particularly sensitive to oxidation and VOC-related flavor deterioration [5,72]. The VOC profile of rabbit meat can be substantially influenced by diet and processing as displayed in Table 5. Supplementation with marine macroalgae (*Ulva* spp.) has been shown to increase MUFA content by 22% without adversely affecting sensory quality [80]. Coffee silver skin has demonstrated antioxidant benefits, improving oxidative stability, although it may slightly reduce ω-3 fatty acid levels. Supplementation with selenium and vitamin E has also proven effective in enhancing oxidative resistance and preserving aroma during storage [81]. Processing conditions play a significant role in flavor retention. Proper chilling post-slaughter (18–24 h) helps stabilize pH and improve tenderness, whereas premature freezing increases drip loss and toughness [82]. Cooking method is another important variable; roasting produces significantly higher aldehyde levels—up to 13-fold—than sous-vide cooking [72]. These approaches offer practical solutions for preserving rabbit meat’s nutritional and sensory quality, aligning with growing consumer interest in lean and sustainable protein sources [83].

### 4.3. VOCs in Eggs

VOCs in eggs, primarily aldehydes, sulfur-containing volatiles and ketones, originate from yolk-derived PUFA oxidation and protein degradation during storage and processing, making them reliable indicators of freshness, sensory quality and production-system identity [42,45]. Their profiles are governed by hen genotype, diet, storage conditions, processing technologies and rearing system, rendering VOC analysis a powerful tool for both product differentiation and shelf-life evaluation (Table 6). Genotype drives breed-specific markers: White Leghorn and Hy-Line Brown eggs both show ~80% aldehyde content, whereas Jing Fen eggs uniquely contain decanal, highlighting potential consumer preference links [42]. Dietary Sacha Inchi oil elevates “nutty” hexanal and nonanal levels but excessive ω-3 enrichment can induce rancidity-related off-flavors, underscoring the need for balanced feed formulations [45]. Under 14-day raw storage of salt-baked marinated eggs, yolk benzaldehyde and 2-methylbutanal decline significantly—more so than in albumen—due to higher yolk PUFA susceptibility [91]. Finally, production system leaves distinct VOC fingerprints: free-range eggs exhibit only eight detected volatiles versus fifteen in caged eggs, while organic eggs feature D-limonene as a citrus-like traceability marker.

## 5. Aromatic Finger Printing

Aroma plays a pivotal role in the quality and consumer acceptance of foods [16]. The complex “fingerprint” of VOCs arising from a food product defines its characteristic flavor and can reveal valuable information about its origin, processing, and freshness. For example, cooked meat aroma is the outcome of interactions among precursors in raw meat undergoing Maillard reactions, peptide pyrolysis, sugar and ribonucleotide degradation, lipid oxidation, thiamine breakdown, and other pathways [16]. The diversity of VOCs is immense—hundreds of compounds spanning classes like aldehydes, alcohols, ketones, esters, acids, furans, sulfur- and nitrogen-containing heterocycles, among others [25]. Each food item or process yields a unique profile of these compounds, analogous to a “fingerprint” that can be used for identification and quality assessment. They can be employed as species-specific, processing-derived and diet-metabolic, food quality and shelf life and traceability/authenticity biomarkers owing to their VOC profiling as discussed below.

### 5.1. Species-Specific Biomarkers

The VOC profile of livestock products is distinctly species-specific due to variations in composition, lipid class distribution, and metabolic pathways. A study by Man et al. [98] demonstrated that VOCs are effective biomarkers for meat species differentiation, showing clear variation in volatile profiles among donkey, bovine, and sheep meats. Key compounds such as hexanal, 1-octen-3-ol, and ethyl acetate were strongly correlated with polyunsaturated fatty acids (PUFAs), particularly in donkey meat, indicating their origin from lipid oxidation. VOCs with high odor activity values (OAVs ≥ 1) and strong correlations with species-specific phospholipids—like PC(O-18:2/20:5)—served as reliable indicators of both flavor and species identity. The composition of VOCs in cooked meat is strongly influenced by species owing to species-specific fatty acid profiling, making them reliable biomarkers for species differentiation. Cooked beef is typically rich in compounds such as octanal, nonanal, (E,E)-2,4-decadienal, methanethiol, methional, 2-furfurylthiol, and 4-hydroxy-2,5-dimethyl-3(2H)-furanone, which contribute to its characteristic meaty-caramel aroma. These compounds also occur in pork and poultry, but at different concentrations. For instance, pork contains lower levels of 4-hydroxy-2,5-dimethyl-3(2H)-furanone due to reduced precursors like glucose-6-phosphate, and a higher ratio of greasy to meaty odorants, such as hexanal and octanal [25]. Poultry meat, on the other hand, is distinguished by VOCs like 2(E)-nonenal, (E,E)-2,4-decadienal, and γ-dodecalactone—products of linoleic acid oxidation [99]. Notably, 12-methyltridecanal, which forms during long stewing of beef, is absent in pork and poultry and contributes to the unique retronasal aroma of beef [4]. In a recent comparative study of beef, pork, chicken, and duck, headspace SPME–GC-MS combined with multivariate statistical analysis revealed differences in volatile profiles [100]. It is important to note, however, that SPME-derived VOC profiles are strongly dependent on fiber coating and extraction conditions and thus represent comparative analytical fingerprints under those specific method settings, rather than absolute volatile compositions [101,102]. Within this method-defined framework, beef was characterized by grassy notes dominated by hexanal and heptanal; pork by sweet and fruity volatiles such as pentan-1-ol and butane-2,3-diol; and poultry by a prevalence of pungent compounds, including certain ketones. A nitrile compound tentatively identified as *3-methylbut-3-enenitrile* was also reported, primarily in duck samples, but this identification remains preliminary without validation using authentic standards or orthogonal methods [100,103]. Despite these methodological constraints, the distinct VOC patterns across species offer valuable relative markers for meat species differentiation, as summarized in Table 7. Table shows that several aldehydes and heterocyclic compounds identified in beef are directly linked to consumer appreciation of roasted and savory flavors. For example, 2-methyl-3-furanthiol and related thiazoles, arising from Maillard and thiamine degradation, are considered key markers of “meaty” and “roasted” aromas that enhance consumer liking. Nonanal and hexanal, derived from lipid oxidation, contribute fatty and grassy notes, respectively; while low levels add desirable complexity, excessive hexanal is perceived as rancid and reduces acceptance. Similarly, furans such as 2-acetylfuran impart caramel-like nuances valued in cooked beef, whereas the accumulation of branched-chain aldehydes may lead to pungent or stale odors disliked by consumers.

### 5.2. Processing-Derived Biomarkers

VOCs are increasingly recognized as effective biomarkers for tracking biochemical changes that occur during processing, such as smoking, ripening, cooking, and curing. These compounds reflect underlying metabolic pathways and ingredient interactions, thereby enabling the differentiation of products based on processing history and authenticity. In a comprehensive study, Yin et al. [104] identified 87 VOCs using GC–MS, with 22 key compounds contributing to aroma as determined by odor activity values. These VOCs—including guaiacol, furfural, hexanal, and 2-methoxy-4-methylphenol—exhibited significant variation depending on the wood type and thus acted as chemical fingerprints for specific smoking treatments. Importantly, the researchers used PCA and partial least squares regression (PLSR) to link VOC patterns with E-nose sensor data. This allowed for clear differentiation between smoked and unsmoked sausages, as well as among sausages smoked with different woods. The high Q^2^ value (0.619) from the PLSR model further supports the predictive power of VOC profiles as discriminant markers [104]. Compounds such as 3-methylbutanoic acid, methional, and phenylacetaldehyde indicate amino acid degradation in ripened sausages [105], while terpenes and sulfur compounds mark seasoning or smoking effects in wet-cured hams [16]. In dry-cured hams, aldehydes and ketones like hexanal and 2-heptanone are characteristic of lipid oxidation and contribute to product-specific aroma profiles [106]. Further, VOCs serve as critical biomarkers for assessing the influence of different cooking treatments on flavor development in processed foods such as golden pomfret. In the study by Chen et al. [72], advanced analytical techniques including GC-MS, GC-IMS, and electronic nose profiling revealed that specific VOCs varied markedly with the applied thermal method, boiling, steaming, microwaving, baking, or air-frying. Aldehydes like hexanal and nonanal, which are products of lipid oxidation, were elevated in steamed and air-fried samples, marking these treatments as oxidative stress-inducing processes. Compounds such as 1-octen-3-ol (mushroom-like) and acetoin (buttery) were more abundant in microwaved samples, suggesting milder degradation of proteins and fats [105].

### 5.3. Diet-Metabolic Biomarker

In the study by Vossen et al. [107], several VOCs were identified as potential biomarkers reflecting both dietary intake and colonic microbial activity in pigs subjected to varied meat types and dietary patterns. Among these, ethyl valerate was significantly more prevalent in pigs fed with red and processed meat, irrespective of dietary pattern. Ethyl valerate and 1-methylthio-propane were more frequently detected in pigs fed red and processed meat, suggesting their role as biomarkers of meat type. The aldehyde 3-methylbutanal was predominantly associated with a Western dietary pattern (>80% prevalence), whereas butanoic acid levels were significantly elevated in pigs fed a prudent diet, indicating distinct microbial fermentation profiles shaped by dietary patterns [107].

### 5.4. Food Quality and Shelf-Life Indicators

Aroma and flavor profiles in animal-derived foods are not only pivotal for sensory appeal but also related to its biochemical composition and shelf-life stability. During processing and storage, lipid oxidation, Maillard and Strecker reactions, and microbial metabolism generate secondary metabolites, e.g., aldehydes, ketones, alcohols, sulfur-containing compounds, that both drive aroma and mirror underlying factors such as fatty-acid profiles and antioxidant levels [23]. Because unsaturated lipids are especially prone to oxidative degradation, their breakdown products serve as indirect indicators of oxidative stability and remaining shelf-life [16].

Milk from organic or pasture-based systems typically contains higher n-3 polyunsaturated fatty acids (α-linolenic and conjugated linoleic acids) and plant-derived polyphenols and terpenes, contributing to “green,” “grassy,” and “floral” aromas as well as enhanced oxidative resistance and nutritional value [108,109]. These compositional traits not only improve flavor but also act as biomarkers of feeding regime and geographic origin. However, batch variability in low-input or pasture-based dairies can be high: forage species (e.g., clover, pasture grasses) and silage inoculants (e.g., arbuscular mycorrhizal fungi) introduce precursors like p-cresol or earthy notes, while fermentation volatiles (acetic, butyric acids) influence both animal intake and milk VOC profiles [110,111]. Genetic factors such as breed choice and crossbreeding, further modulate these effects, underscoring the challenge of achieving consistent milk aroma and flavor in sustainable dairy systems [112].

### 5.5. Authenticity/Traceability Markers

Ensuring authenticity in animal-derived foods demands robust analytical tools for verifying species, origin, and production systems. Traditional genetic and chemical markers, e.g., DNA barcoding (COI gene), PCR-RFLP, remain cornerstones for species identification, with full-length barcodes (658 bp) generally outperforming mini-barcodes (127 bp) except in highly processed products where DNA degradation favors shorter targets [113]. Emerging molecular methods (droplet digital PCR, isothermal amplification, CRISPR/Cas) further enhance precision.

Traceability systems now integrate isotope fingerprinting (δ^13^C, δ^15^N, δ^2^H, δ^18^O, δ^34^S), spectroscopy, and blockchain to confirm geographic origin and production transparency [114]. Stable-isotope ratio analysis (SIRA) can distinguish dairy regions with up to 100% accuracy (δ^13^C + δ^18^O) in model products and multi-isotope profiles classified samples across Ireland, Europe, Australasia, and the USA at 88% accuracy using random forests [115]. Complementary studies in Sri Lanka and Slovenia combine isotopes with trace-element fingerprints and multivariate models to robustly differentiate agro-climatic zones [116,117]. Differentiating organic, halal, and kosher systems increasingly relies on metabolomic profiling, stable isotopes, and biomarker assays, with chromatographic (HPLC, GC), spectroscopic (FTIR, NIR, Raman), and molecular techniques detecting prohibited residues, while machine learning–driven proteomics reveals unique protein signatures linked to specific farming and processing practices [118]. These advanced tools enhance the accuracy and robustness of food authentication, supporting both consumer trust and regulatory compliance in value-driven product categories. Thus, aromatic profiles serve not only as quality indicators but also as traceability tools in authenticity assurance systems.

## 6. Role of Aroma-Active VOCs in Consumer Perception and Market Behavior

Beyond their role in defining the sensory characteristics of animal-derived foods, aroma-active VOCs have increasingly been recognized as influential factors in shaping consumer perception and driving market behavior. These compounds, responsible for the distinctive aroma and flavor of meat, milk, and eggs, can affect not only how products are evaluated during consumption but also how they are perceived prior to purchase, often influencing consumer trust, preferences, and willingness to pay. As modern consumers seek products that align with values such as quality, authenticity, sustainability, and health, the sensory experience, anchored by aroma and flavor, has become a critical touchpoint in the decision-making process.

This section explores the evolving role of VOCs in consumer-oriented dimensions of food science focusing on how specific aroma and flavor attributes influence sensory evaluation and consumer choice. It discusses the importance of authenticity cues and quality labels in shaping purchasing behavior and market segmentation and addresses the broader impact of socio-cultural and market trends on consumer preferences, highlighting the interplay between flavor perception, cultural identity, and emerging values in the global food landscape.

### 6.1. Influence of Aroma and Flavor on Sensory Evaluation and Purchasing Decisions

Aroma and flavor are pivotal to consumer acceptance, strongly influencing both initial selection and repeat purchases of dairy and meat products. Sensory evaluation remains the most definitive method for assessing product quality, particularly in detecting key VOCs that shape consumer perception through aroma and off-flavor profiles [50]. In dairy products, specific VOCs such as aldehydes, ketones, and lactones contribute significantly to perceived freshness and flavor intensity, with certain compounds acting as markers of both positive and negative sensory attributes [50]. Consumer preference often correlates more strongly with desirable flavor characteristics, such as milky and creamy notes, than with off-flavors, reinforcing the role of congruent sensory experiences in product appeal [66]. Additionally, diet, processing, and formulation practices impact VOC formation and thus sensory perception, underlining the need for integrated sensory-analytical approaches in product optimization [50,119].

Consumers often associate pasture-fed dairy and beef products with more “natural” and “healthier” profiles, largely influenced by their distinct organoleptic characteristics such as herbal, gamey, or grassy flavor notes [120]. However, these sensory qualities are not universally accepted; in regions where consumers are more familiar with neutral or sweeter profiles, such as in parts of Asia, these same attributes may be perceived as off-flavors [121,122]. This highlights the importance of cultural context in shaping flavor perception and market acceptance. Despite well-documented nutritional advantages of pasture-based feeding systems, including elevated levels of beneficial fatty acids (e.g., CLA, omega-3), antioxidants, and fat-soluble vitamins [123], consumer premiums are not always captured by producers due to a lack of recognized differentiation in grass-fed dairy and meat products [122]. Notably, recent studies highlight that Chinese consumers, for instance, show greater willingness to pay for tangible naturalness attributes such as grazing conditions and grass-based feeding, rather than abstract imagery like health or sustainability claims [122]. This suggests that effective product differentiation strategies should be grounded in verifiable production traits rather than marketing narratives alone. Ultimately, understanding regional flavor preferences and aligning product communication accordingly is critical to unlocking the value of pasture-based systems in global markets.

### 6.2. Role of Authenticity and Quality Claims in Consumer Behavior and Market Segmentation

Quality labels and origin claims such as organic, grass-fed, halal, or PDO (Protected Designation of Origin) are significant drivers of consumer trust and purchasing behavior in food markets. These credence attributes, which consumers cannot directly verify even after consumption, influence decisions by aligning with ethical, environmental, and health-related values [124]. Multiple international studies demonstrate that consumers increasingly value verified claims, associating them with superior food quality, safety, sustainability, and animal welfare [125]. For example, European consumers exhibit a strong preference for national origin and organic labels, often willing to pay a premium for such attributes [126]. Similarly, legitimacy perceptions, particularly pragmatic, moral, and regulative strongly shape purchase intentions for PDO-labeled products in France [125]. However, the effectiveness of labels depends on consumer awareness, cultural context, and perceived label credibility [127]. In fragmented markets or where consumer knowledge is limited, the proliferation of labels can cause confusion, underscoring the need for standardization and clearer communication [127]. Furthermore, digital marketing strategies targeting identified consumer segments have shown promise in raising awareness and influencing behavior towards certified products, as evidenced in the Romanian market [128]. Globally, even in emerging markets like China, credence attributes of organic food positively affect attitudes and willingness to pay a premium, moderated by factors such as perceived uncertainty [128]. Overall, verified quality and origin claims serve as effective market signals, shaping distinct consumer segments and supporting price premiums in competitive food systems.

The alignment between marketing claims and sensory expectations plays a critical role in maintaining consumer trust and fostering repeat purchasing behavior, particularly within food systems. When consumers encounter a discrepancy between the promoted sensory attributes and their actual experience, such as expecting creamy mildness in an organic cheese and instead perceiving overpowering barnyard flavors, this misalignment can undermine perceived product authenticity and erode brand credibility. Prior research emphasizes that such inconsistencies can significantly reduce repurchase intent and consumer loyalty. For instance, a study on Chinese consumer trust in the domestic dairy sector revealed that trust remains fragile following the 2008 melamine scandal, with consumer beliefs about actor competence and transparency continuing to affect perceptions of product integrity [129]. Similarly, the interplay between sensory experience and labeled information has been shown to influence purchasing intentions. In fresh milk, positive sensory perceptions, especially when congruent with health- and nutrition-oriented labels, mediate the relationship between product information and consumer intention to buy, highlighting the importance of sensory validation in consumer decision-making. The critical role of expectation alignment is also evident in plant-based cheese alternatives, where systematic negative expectations related to flavor and texture led to consistently lower consumer appeal compared to dairy counterparts, despite shared conceptual attributes. Hence, misalignment between sensory experiences and marketing claims not only disrupts consumer satisfaction but also compromises trust and long-term brand engagement, particularly in sectors where product integrity and experiential fidelity are paramount. While sensory studies are essential for linking aroma-active VOCs to consumer preferences, they remain constrained by subjectivity, panel variability, and context dependence. Regional diversity in flavor expectations also means that labels such as “grass-fed,” “organic,” or “PDO” may be interpreted differently across markets, underscoring the need for region-specific validation to maintain authenticity and trust.

### 6.3. Market Trends and Sociocultural Factors Shaping Preferences

Consumer preferences are evolving under the influence of sustainability, clean-label and ethical-consumption trends, yet remain firmly grounded in cultural heritage and regional traditions [130]. Globalization has expanded exposure to diverse ethnic flavors and fusion cuisines, but longstanding dietary norms still prevail—for example, strong fermented dairy is favored in Scandinavian and Eastern European markets [131], whereas mild, subtly sweet profiles dominate East Asia. In Sri Lanka, *Meekiri* (fermented buffalo milk gel) enjoys widespread socio-economic importance despite scant scientific study of its properties [132]. Mediterranean and Atlantic dietary patterns—plant-rich yet dairy-inclusive—are lauded for health and environmental benefits over meat-heavy Western diets [106]. Meanwhile, meat-reduction and plant-based innovations driven by sustainability and animal-welfare concerns hinge on perceived naturalness, healthfulness and authenticity for consumer acceptance [130,133].

Sociodemographic factors—age, education, income and dietary ideology (e.g., vegetarianism, flexitarianism, religious observance)—further segment these evolving markets by shaping individual attitudes and decision-making [134]. To meet these varied demands, manufacturers blend sensory optimization [135] and ethical marketing with Industry 4.0 tools—blockchain, smart labels and precision systems—to enhance traceability, sustainability and agility [136]. This approach highlights the value of co-creation and cultural insight in developing novel products that resonate with—and dynamically adapt to—diverse global consumer segments [134].

## 7. Aromatic Fingerprinting Techniques

Given the complexity of aroma profiles, analytical techniques are essential to separate, identify, and quantify VOCs more efficiently and objectively than human sensory panels. Gas chromatography–mass spectrometry (GC–MS) has long been the gold standard for VOC analysis, capable of separating individual volatiles and identifying them via their mass spectra [137,138]. However, newer and complementary approaches have emerged to capture holistic aroma fingerprints. Electronic noses (E-nose), which employ arrays of non-specific chemical sensors, provide rapid “smell prints” of headspace gases without the need to isolate each compound [105]. Although E-nose is a valuable technique to distinguish samples and the magnitude of difference among samples, it does not indicate a specific VOC and their aroma contribution so the results obtained should be interpreted with care. Meanwhile, spectroscopic methods, broadly including techniques like ion mobility spectrometry and infrared spectroscopy, can generate distinctive spectral patterns for VOC mixtures, offering quick fingerprinting. The aromatic fingerprinting techniques for profiling volatile compounds, with a focus on GC–MS, electronic noses, and spectroscopic methods, have been detailed in subsequent section along with biochemical sources and biogenesis of VOCs, and their relevance as biomarkers for quality and authenticity. Further, recent innovations in instrumentation and data analysis that enhance the sensitivity and specificity of aroma profiling have also been highlighted.

### 7.1. Analytical Techniques for Aromatic Fingerprinting

The principle underlying VOC analysis involves several systematic stages: initially, VOCs are sampled from the environment through either active collection, where air is actively drawn through a sorbent, or passive collection, relying on natural diffusion driven by concentration gradients. These sampled VOCs are subsequently enriched and concentrated onto solid sorbent materials, typically inorganic sorbents, porous carbon-based materials (such as activated charcoal or graphitized carbon black), or organic polymer-based sorbents. Following enrichment, the VOCs are released from the sorbent by either thermal desorption, which uses controlled heating to enhance volatility, or solvent extraction, which leverages differences in compound solubility between water and organic solvents. The released VOCs are then transferred to specialized analytical instruments, where they undergo precise detection and identification [139].

Food aroma analysis relies on an array of analytical tools to capture the complex mixture of VOCs. Traditional GC–MS provides detailed compound-specific information, whereas newer approaches like E-noses and spectroscopic sensors yield rapid overall fingerprints. Combining these techniques can offer both breadth and depth in VOC profiling [140]. Below, we outline the principles and applications of GC–MS, electronic noses, and spectroscopic methods in aromatic fingerprinting.

#### 7.1.1. Gas Chromatography–Mass Spectrometry (GC–MS)

GC–MS is widely regarded as the benchmark for volatile analysis and has been extensively used to separate and identify aroma compounds in foods [141]. GC–MS effectively integrates the strengths and capabilities of gas chromatography (GC) and mass spectrometry (MS). Initially, volatile molecules are separated using a capillary GC column based on their boiling point and polarity, forming distinct retention times depicted as peaks in a chromatogram. Upon entering the mass spectrometer, these separated components are captured, ionized, and detected according to their mass-to-charge (m/z) ratios, providing a characteristic mass spectral “fingerprint” for precise identification of each compound [142,143]. This technique excels in resolving complex mixtures of VOCs, allowing the detection of dozens to hundreds of compounds from a single sample. One significant advantage of GC–MS lies in its remarkable sensitivity and its ability to provide structural information, enabling analysts to precisely pinpoint specific aroma molecules even at extremely low concentrations. Many flavor-relevant VOCs, such as sulfur compounds or certain aldehydes, have notably low odor thresholds, and GC–MS reliably detects these compounds at concentrations down to parts-per-billion levels. By matching mass spectra against established databases and retention indices, compounds can be effectively identified or tentatively characterized [144,145,146]. As a practical example, hexanal, a predominant aldehyde derived from fatty acids, was clearly identified through GC–MS in goat meat VOC analyses [147]. Similarly, GC–MS analysis of smoked sausage revealed 87 distinct volatiles, including guaiacol and 2-methylphenol, compounds directly linked to wood smoke treatments [104]. However, GC–MS analysis is relatively time-consuming (a typical run may take tens of minutes), requires sample preparation (such as HS-SPME enrichment of headspace) [105], and the instrumentation is costly and requires expertise to operate. Some highly volatile or reactive compounds can also be challenging to trap and analyze. Innovations to address these issues include fast-GC or two-dimensional gas chromatography mass spectrometry (GC × GC-qMS) for quicker or higher-resolution separations, and improved detectors or high-resolution mass spectrometers for greater sensitivity [148]. Nonetheless, GC–MS remains the cornerstone for confirming identities of key aroma compounds and quantifying them. Often, it is used in tandem with fingerprinting methods: for example, an E-nose might rapidly screen samples for differences, and then GC–MS is employed to pinpoint which specific compounds differ.

#### 7.1.2. Electronic Noses (E-Nose)

E-nose have emerged as powerful tools for VOC fingerprinting, particularly in the assessment of meat freshness, authenticity, and spoilage detection [149,150]. These devices employ sensor arrays—commonly semiconductor metal-oxide sensors (MOS), conducting polymers (CP), or quartz crystal microbalances (QCM)—that respond to volatile compounds by altering electrical resistance or frequency, generating characteristic “smell prints” that can be analyzed with chemometric or machine learning tools [105,151]. While such fingerprints enable robust sample discrimination and quality monitoring, they do not directly equate to human aroma perception. Nonetheless, correlations with sensory evaluation have been reported, such as the strong relationship between e-nose signals and cheese aroma intensity scores [152], as well as links between sensor responses and diet-induced changes in lamb meat volatiles [153]. Meat applications remain the most extensively studied, with e-noses consistently detecting spoilage-related VOCs such as dimethyl sulfide, trimethylamine, hexanal, methanol, ethanol, and methyl thioacetate [154,155,156]. A recent advance is the development of a microcantilever-based system functionalized with a cadaverine-selective binder, which correlated e-nose responses with bacterial counts and biogenic amine levels in poultry, providing accurate shelf-life estimations that aligned with microbial and sensory data [157]. E-noses are also used for species authentication and adulteration detection; Nurjuliana et al. [158] successfully discriminated sheep, cattle, poultry, and swine, while Tian et al. [159] quantified pork adulteration in minced mutton using MOSs combined with PCA, linear discriminant analysis (LDA), and artificial neural networks (ANNs). More recently, compact e-nose systems integrated with supervised learning achieved >99% accuracy in classifying meat floss samples and identified pork-specific aldehydes such as dodecanal and 9-octadecanal, underlining the potential for fraud detection and dietary compliance monitoring [160]. Beyond authentication, e-noses have been applied to monitor dietary supplementation effects, with antioxidant-rich feeding strategies altering meat VOC patterns in ways reliably detected by e-nose analysis [153,161], and to identify quality defects such as boar taint and warmed-over flavor [162,163]. E-noses have been successfully applied in dairy systems for rapid quality assessment. Ref. [164] used an e-nose combined with an e-tongue to distinguish milk quality and brand differences over storage time. In cheese, Fujioka [152] found a remarkably high Pearson’s R of 0.983 between e-nose signals and sensory panel aroma intensity. These applications reinforce the role of e-noses as complementary rapid-screening tools, which can be followed by chromatographic methods for compound-specific validation. Recent technological advances have enhanced both performance and integration. Studies combining e-nose data with GC–MS have shown that specific sensor outputs can be quantitatively linked to volatiles such as 1-octen-3-ol in smoked meat products [104]. IoT-enabled e-nose systems that integrate CO_2_, NH_3_, and ethylene sensors with wireless cloud-based monitoring have demonstrated utility for real-time beef freshness tracking under varying storage conditions [165]. Sensor innovations, including microcantilever functionalization for biogenic amine detection [157], have further improved sensitivity and specificity to key spoilage markers. Despite these promising advances, e-noses face challenges that limit industrial adoption. Their lack of specificity for individual compounds, susceptibility to drift and environmental effects such as humidity, and need for calibration with standardized datasets remain barriers [150]. Nevertheless, when coupled with confirmatory techniques like GC–MS or GC–IMS, E-noses provide rapid and non-destructive VOC profiling. This complementary role—fast pattern recognition by e-nose followed by detailed compound identification through chromatography—represents a pragmatic strategy for quality control in modern food supply chains [16,105].

### 7.2. Spectroscopic and Emerging Sensor Methods

Spectroscopic methods offer alternative ways to profile volatile compounds by measuring their interaction with electromagnetic radiation or other physical fields, often yielding a characteristic spectrum or pattern. These techniques can be powerful for rapid fingerprinting and have the benefit of being reagentless and often requiring minimal sample preparation. Key examples include infrared spectroscopy (especially Fourier-transform infrared, FTIR, in the mid-IR region) and ion mobility spectrometry (IMS). We also consider GC-IMS hybrids and other novel instrumentation that improve VOC detection.

#### 7.2.1. FTIR Spectroscopy

FTIR spectroscopy has demonstrated its utility as a rapid, reliable, and non-invasive tool to assess meat spoilage by analyzing the unique vibrational signatures of functional groups in organic molecules for real-time [166,167]. These spectral fingerprints provide a comprehensive biochemical profile of meat, which can be analyzed through chemometric tools to determine spoilage status or microbial load. The efficacy of FTIR in detecting VOCs and microbial metabolites is attributed to its sensitivity in specific spectral regions. Absorbance bands between 3000 and 2800 cm^−1^ represent C–H stretching of fatty acids; 1700–1500 cm^−1^ corresponds to Amide I and II bands from protein degradation; and 1200–900 cm^−1^ captures carbohydrate vibrations from microbial cell wall polysaccharides [168]. These regions are highly relevant for spoilage detection, as microbial contamination leads to protein denaturation, lipid hydrolysis, and formation of specific VOCs such as aldehydes, ketones, and organic acids. FTIR spectroscopy has been successfully applied to differentiate between fresh and spoiled meats. For instance, Ellis et al. [169] employed FTIR in combination with machine learning tools like genetic programming (GP) and PLSR to predict spoilage in comminuted chicken breast. The Amide II band (around 1550 cm^−1^) showed a negative correlation with microbial growth due to declining protein content, while absorbance at 1240–1088 cm^−1^, indicative of free amino acids, increased as spoilage progressed. Similarly, Amamcharla et al. [170] used FTIR coupled with a gas cell for headspace VOC analysis of beef contaminated with *Salmonella*. PCA revealed that the region from 850–500 cm^−1^ could effectively discriminate contaminated from uncontaminated samples, which suggested that FTIR can capture microbial volatile markers, thereby facilitating early spoilage detection In other study, Zajac et al. [171] used time-resolved FTIR to monitor protein degradation in chicken meat during storage. They identified shifts in the Amide I, II, and III bands and S–S stretching as reliable indicators of spoilage, correlating well with increased free amino acid content and microbial activity. FTIR spectroscopy has also been utilized to characterize spoilage in dry-fermented sausages during storage at different temperatures, showing excellent correlation between microbial loads and IR spectral features related to peptides and saccharides [172]. The predictive accuracy of FTIR spectroscopy can be enhanced by coupling with machine learning algorithms such as support vector machines (SVM), ANNs, and adaptive fuzzy logic systems (AFLS) for spoilage classification across different meat matrices [168].

#### 7.2.2. Ion Mobility Spectrometry (IMS)

IMS is a technique that separates ionized molecules in the gas phase based on their size, shape, and charge by measuring their drift time through a tube under an electric field and a buffer gas flow. In essence, IMS provides a “mobility spectrum” of volatile compounds. Coupling IMS with GC (GC–IMS) has gained traction for volatile analysis in foods, as it combines a modest chromatographic separation with a secondary separation in the IMS drift tube. A GC–IMS instrument generates a 2-dimensional output: retention time (GC) vs. drift time (IMS), often visualized as a topographic plot or a heatmap “fingerprint” for each sample [105]. Notably, GC–IMS can detect compounds at low concentrations (down to ppb levels) and operates at atmospheric pressure, making it suitable for rapid on-site analysis. It has been described as a “new technique for hot gas phase separation detection” with the ability to characterize volatiles at the molecular level [16]. In the study of Chen et al. [72], GC–IMS was employed alongside GC–MS and E-nose to profile fermented fish aroma under different cooking methods. The GC–IMS generated distinct 2D spectral fingerprints for each cooking treatment. In Chen et al.’s results, the number and intensity of volatile features differed markedly between cooking methods, and a total of 72 volatiles were detected by GC–IMS across all samples. This allowed a quick visual comparison: for instance, some volatiles (like 2-hexanone, ethyl sulfide, 2-methyl-2-pentenal) appeared in all samples (common aroma components), whereas others were unique or significantly more abundant in one cooking method versus another. Such IMS fingerprints can act as characteristic signatures of a product’s volatile profile and are increasingly used for authenticity testing and quality control. The technique combines high sensitivity with short analysis times, as a typical GC–IMS run is often completed within 10–15 min. Beyond flavor characterization in cooked meats, several primary studies have demonstrated its value in detecting food adulteration. For instance, GC–IMS has been applied to grilled lamb products, where differences in volatile fingerprints revealed undeclared formulation changes [173]. Likewise, regional meat products such as Chinese bacon have been differentiated by GC–IMS, providing a practical approach to identify origin mislabeling and potential fraud [174]. These applications illustrate how IMS-based volatile profiling moves beyond general aroma evaluation to offer direct evidence for authenticity and adulteration in specific food categories. Additionally electronic tongues (E-tongues), though not designed to detect VOCs directly, are increasingly used alongside GC–MS, GC–IMS, and E-noses to provide complementary information on non-volatile taste-active compounds. Such integrated approaches have been applied in beef and sheep meat studies, linking E-tongue outputs with VOC profiles for a more comprehensive flavor characterization [175,176,177]. Further, to facilitate comparison, Table 8 summarizes the main aromatic fingerprinting techniques, highlighting their detection principles, typical detection/quantification limits, advantages, and limitations.

## 8. Conclusions

This review demonstrates that VOC profiles in meat, milk, and eggs are fundamentally shaped by farm management practices including feeding strategies, animal breed, housing systems, and post-farm processing operations. VOCs arising from lipid oxidation, Maillard reactions, microbial metabolism, and other biochemical pathways not only govern sensory attributes but also serve as robust biomarkers of food quality, production authenticity, and consumer perception. Comparative studies reveal system-dependent signatures elevated aldehydes and terpenoids in grass-fed meat, enhanced polyphenols and sulfur compounds in pasture-based milk, distinct breed- and housing-dependent egg aromas. High-resolution platforms such as GC–MS, GC–IMS, FTIR spectroscopy, and electronic-nose systems enable precise VOC fingerprinting for quality assurance, authenticity testing, and the design of sensory-driven, value-added foods. Looking forward, integrating VOC profiling with genomics, metabolomics, and machine learning may offer real-time prediction of sensory outcomes and traceability across diverse production systems. To realize this potential, future research must standardize VOC biomarkers, advance rapid on-site detection tools, and rigorously validate multi-omics flavor models through statistically robust methods. However, the outcomes from fundamental and applied research associated with on farm feeding practices on the development of VOC profile in animal-derived products cannot be overlooked as it is an integral part of VOC development. These outcomes should always be connected to the findings of genomics, metabolomics, and machine learning. Such a multidisciplinary strategy will foster transparency and sustainability in the food chain while aligning animal husbandry with evolving consumer demands for nutritious, ethically produced, and sensorially compelling foods. This review provides following key recommendations:

- Standardize VOC biomarkers and analytical protocols so results are comparable across systems and studies.
- Advance rapid, on-site detection tools (e.g., portable instruments, sensor platforms) to enable real-time monitoring in production chains.
- Validate multi-omics flavor models (linking VOCs with genomics and metabolomics) through robust statistical approaches for practical application.
- Connect farm-level feeding practices with advanced omics to ensure that fundamental nutritional effects on VOC development are not overlooked in high-tech models.

Together, these steps can turn VOC research into practical tools that connect farm practices with consumer trust, ensuring animal-derived foods remain authentic, high-quality, and transparent.

## 9. Future Perspectives and Research Directions

**Figure 4:**
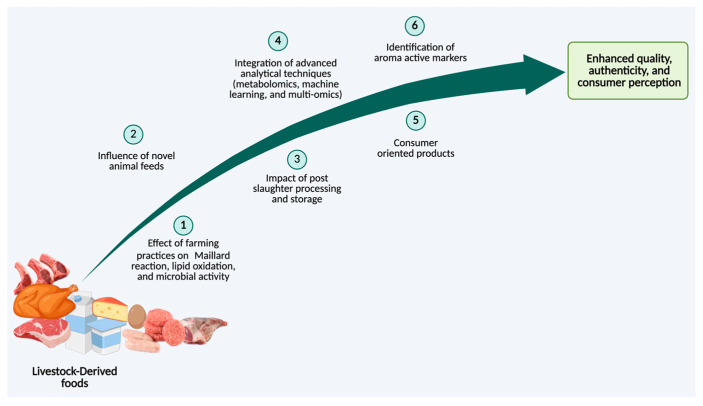
Future perspective and research direction for farming practices and aromatic fingerprints of animal-derived foods.

The integration of aromatic fingerprinting into animal-derived foods is a pivotal advance for improving quality, nutrition, authenticity, and consumer trust (Figure 4). Databases will enable scalable, evidence-based quality control in these products. Although this review is developed for terrestrial foods, extending the same framework to seafood of nutritional interest will test transferability, expand marker libraries, and support unified standards for provenance and label integrity.

Beyond current links between farming practices and product quality, deeper resolution is needed on how diet composition (grass vs. grain, feed additives), production systems (indoor vs. free-range), antibiotic use, stress, and housing reshape meat, milk, or egg composition and modulate metabolic pathways (Maillard reactions, proteolysis, lipid oxidation, microbial activity). Future studies should use simple and multiple regression to relate VOC formation to sensory attributes and identify aroma-active markers for freshness, spoilage, nutrient loss, production method, and origin. A practical research agenda is required to integrate GC-MS or electronic-nose outputs with machine learning and multi-omics under standardized, internationally validated protocols with adequate sample sizes, interlaboratory reproducibility, and physiological and biochemical cross-checks. Priority gaps include processing effects (pasteurization, fermentation, aging) and resident microbiomes, especially in raw milk and dry-aged meat, as well as the long-term influence of novel feeds such as seaweeds, agro-byproducts, and insect meals. Translation will benefit from aroma mapping aligned to consumer research, longitudinal farm-to-fork cohorts capturing seasonal and environmental drivers, and deployment of rapid on-site diagnostics with digitally verifiable traceability. Finally, developing rapid on-site diagnostic tools and blockchain-enabled tracea-bility systems will help align optimized feed formulations and reduced antibiotic use with animal health, muscle growth, and milk/meat quality thereby establishing a ho-listic framework for flavor-driven sensory quality control in animal-derived foods.

## Figures and Tables

**Table 1:** Factors influencing volatile organic compound profiles in milk and dairy products.

Factor	Food Matrix	Key VOCs	Effect on VOC Profile	Reference
Dairy system (TMR vs. separate feeds; herd, lactation stage)	Cheese, Milk	Alcohols, esters ↑; Acetic acid ↓	TMR increased fruity notes; silage-based TMR reduced overall volatiles; days in milk significantly influenced VOC patterns	[46]
Feeding system (Rangeland vs. Indoor)	Milk	Terpenes (unspecified)	Rangeland milk showed terpene variations, though specific compounds were not detailed	[49]
Grazing vs. Indoor feeding	Milk	Aldehydes, terpenes, sulfur ↑ (grazing); Ketones (acetone), acids (hexanoic, octanoic) ↑ (indoor)	Clear diet-dependent segregation of VOC classes	[48]
Fresh forage inclusion (Sorghum vs. silage)	Milk, Cheese	Aldehydes (green notes) ↑; Ketones, acids, esters (fruity/cheesy notes) ↑	Forage enhanced green notes; silage increased fruity/fermented notes	[48]
Breed (Merino, Lacaune, Assaf)	Milk	Ketones (Merino: 71.8%); Hydrocarbons (Lacaune: 37.2%; Assaf: 55.4%); Acetone correlated with *Salinicoccus*, *Psychrobacter*	Breed-specific microbial–VOC associations evident	[9]
Diet (Whole vs. ground flaxseed)	Milk	Aldehydes (nonanal ↑); Fruity/sweet VOCs ↓	Whole flaxseed altered 22 VOCs; ground flaxseed only 5 VOCs altered	[30]
Diet (Soybean meal, Yellow wine lees, Fermented lees)	Milk	PCA-based VOC differences (specific compounds not detailed)	Distinct diet-dependent VOC patterns identified	[30]
Diet (Grass, Grass/clover, TMR)	Milk powder	1-Pentanol, 1-Hexanol	Levels varied significantly across diets, influencing sensory attributes	[50]
Herbal feed additives	Milk	Caproic (C6:0), Caprylic (C8:0), Capric (C10:0) acids ↓; Methyl ketones (2-heptanone, 2-nonanone) ↑; Esters ↑	Reduced “goaty” smell; enhanced fruity/creamy notes	[51]
Diet (Jujube supplementation)	Milk	PCA correlations with VOCs (specific VOCs not detailed)	Jujube supplementation altered serum–VOC correlations	[52]

VOCs = Volatile organic compound; ↑ = Increase; ↓ = Decrease; PCA = Principal component analysis; TMR = Total mixed ration; C6:0 = Caproic acid; C8:0 = Caprylic acid; C10:0 = Capric acid.

**Table 2:** Effects of breed and diet on beef quality.

Breed	Feed/Duration	Key Flavor and Aroma Compounds	Sensory Qualities	Key Findings	References
Late maturing suckler steers	Barley-based concentrate (97 days)	Increased Maillard-derived compounds	↑ Tenderness, IMF ↑ cooking loss	Grain-finishing enhances marbling but may reduce juiciness.	[56]
Crossbred steers	Benzoic acid (0.5% DM, 98 days)	Enhanced beefy, roasted notes	Stronger beef flavor, no texture differences	No impact on shear force or oxidation.	[54]
Holstein-Friesian × Limousin	Grass silage + concentrate (18 months)	Higher aldehydes (hexanal, nonanal)	Bulls: Leaner, less tender; Steers: Juicier	Gender affects tenderness more than diet.	[57]
Grass-fed vs. grain-fed	Pasture vs. concentrate (~100 days)	Grass-fed: Grassy (hexanal); Grain-fed: Roasted (nonanal)	Grass-fed: ↓ Tenderness, ↑ oxidation stability	Grain-fed preferred for “beefy” flavor.	[58]
Canchim steers (5/8 Charolais × 3/8 Zebu)	Pellet diet (peanut shell, corn, soybean meal), dry-aged 28 days	Methional (cheddar cheese), furan (roasted beef)	Enhanced tenderness, preferred flavor	Dry aging increased tenderness and unique volatile compounds.	[53]
Charolais cull cows	RM-1: Mostly pasture-fed, low concentrate	-	↓ Flavor intensity, ↓ fat aroma	Yellower fat, smoother meat grain.	[59]
Charolais cull cows	RM-3: High concentrate, mainly housed	-	↑ Flavor intensity, ↑ fat aroma	Stronger but sometimes atypical flavors.	[59]

↑ = Increase; ↓ = Decrease; IMF = Intramuscular fat; DM = Dry matter; RM-1 = Pasture-based diet with low concentrate; RM-3 = High-concentrate diet.

**Table 3:** Effects of breed and diet on aromatic and sensory profiles in sheep and lamb meat.

Breed	Feed/Duration	Key Flavor and Aroma Compounds	Sensory Qualities	Key Findings	Reference
Texel × Scottish Blackface lambs	Silage vs. concentrate finishing	↑ Lamb aroma (concentrate); manure/fecal notes (silage)	Silage: off-notes reduced by mixed diets	Mixed diets reduce negative sensory traits	[35]
Gallega Iberian lambs	Silage vs. concentrate (4–4.5 months)	↑ Hydrocarbons and aldehydes (concentrate)	Grass-fed: benzyl alcohol marker	Concentrate increases aldehydes; grass-fed retains pasture markers	[63]
Tan sheep	Mixed grazing + indoor (90 days)	↑ Pleasant volatiles (ketones)	↑ IMF, juiciness	Mixed systems optimize flavor and tenderness	[64]
Santa Inês lambs	Rehydrated corn silage	Not specified	Improved tenderness and balanced aroma	Complete corn replacement feasible with no carcass penalty	[65]
Crossbred lambs	Yeast culture (1.0%, 60 days)	↑ 2-decenal (E), nonanal	Higher IMF, reduced cooking loss	Increased oleic acid and redness (a*)	[66]
Merino × Dorper lambs	Microalgae (0.5–1% DM, 98 days)	↑ ALA and omega-3 LC-FAs	↑ Drip loss at 0.5%	1% DM reduced IMF; no impact on growth	[67]
Small-Tailed Han sheep	Ensiled protein grass (8 weeks)	Citrus-like aldehydes	↑ Omega-3, diversified aroma	Improved fatty acid profile and aroma complexity	[68]

↑ = Increase; IMF = Intramuscular fat; DM = Dry matter; LC-FAs = Long-chain fatty acids; ALA = Alpha-linolenic acid. a* = Redness index in colorimetry.

**Table 4:** Effect of different factors on the VOC profile and sensory qualities of poultry meat.

Species/Breed	Factor (Feed/Age/Environment)	Key VOCs (Examples)	Positive Attributes	Negative Attributes	Reference
Ross 308 broilers	Black cumin seed meal (20–60 g/kg)	Pyrazines, aldehydes ↑	Improved aroma, reduced drip loss, better protein and color	–	[69]
Daheng broilers	Age (60–180 days)	Hexanal, 1-octen-3-ol	Higher IMF, richer flavor at 150 days	Slightly higher oxidative products with age	[41]
Native Chinese chickens	L-glutamine supplementation	Nonanal, hexanal ↑	Enhanced umami and Maillard aromas	–	[73]
Arbor Acres broilers	Epigallocatechin gallate (750 mg/kg)	Flavor amino acids ↑	Improved antioxidant capacity, reduced drip loss, lighter color	–	[74]
White-Feather broiler	Fermented coffee pericarp (2.5%)	Aldehydes, ketones, alcohols, esters ↑	Enhanced aroma, reduced drip loss, higher protein	–	[71]
Broiler chickens	Housefly larva meal (5%)	Sulfurous thiols	Higher flavor desirability, sustainable protein source	–	[70]
Jingfen laying hens	HELP diet (model group)	Fruity, waxy, tropical VOCs	–	Reduced tenderness, higher cooking loss, lower pH	[75]
Turkeys	Blue lupine meal (180 g/kg)	Not specified	Improved weight gain	Increased breast hardness	[76]
Egyptian goose	Seasonal diet (winter vs. summer)	PUFA volatiles (winter), MUFA volatiles (summer)	Summer diet: sweet-oily mild aroma	Winter diet: strong gamey aroma	[77]
Japanese quail	Garlic powder (1%)	Reduced oxidation (TBA/peroxides)	Improved stability, best sensory score at 1%	–	[78]
Laying hens	Sacha inchi oil (0.5%)	ω-3 PUFA ↑, improved ω-6/ω-3	Healthier fatty acid profile, stronger desirable flavor	Potential oxidative susceptibility at high ω-3	[45]
Pigeon (squabs)	DL-methionine (30–120 mg/kg)	Not specified	Improved tenderness, higher yield	–	[79]

↑ = Increase; IMF = Intramuscular fat; MUFA = Monounsaturated fatty acids; PUFA = Polyunsaturated fatty acids; ω-3/ω-6 = Omega-3/omega-6 fatty acid ratio; VOCs = Volatile organic compounds; TBA = Thiobarbituric acid (measure of lipid oxidation); HELP diet = High-efficiency low-pollution diet; pH = Measure of meat acidity; cooking loss = Moisture loss during cooking; DL-Methionine = Synthetic methionine supplement; g/kg = Grams per kilogram; mg/kg = Milligrams per kilogram.

**Table 5:** Key influencing factors of volatile organic compounds in rabbit meat.

Factor Investigated	Key Findings on VOCs and Meat Quality	Reference
Diet		
Marine macroalgae (*Ulva* spp.)	Increased fat content (0.96% vs. 0.33% control) and MUFA by 22%. No effect on moisture, protein, or ash. No negative sensory impact.	[80]
Coffee silverskin (CSS)	Reduced ω-3 fatty acids but improved oxidative stability (lower TBARS). No change in total SFA/MUFA/PUFA.	[84]
Flaxseed oil (FSO) + antioxidants (ALC, LCO, PCA)	Increased ω-3 content but required antioxidants to prevent oxidation. Punicalagin showed the strongest antioxidant effect.	[85]
Selenium (Se) + Vitamin E	Organic Se + Vitamin E improved PUFA content and oxidative stability (lower MDA). Higher Se deposition in muscles than inorganic Se.	[81]
Processing and storage		
Chilling time (18–24 h)	Reduced thawing losses, improved tenderness, and stabilized pH. Rigor mortis resolved by 18 h, enhancing meat quality.	[82]
Freezing vs. chilling	Freezing pre-rigor meat increased exudate loss and toughness. Optimal chilling (18 h at 4 °C) before freezing improved quality.	[86]
Irradiation (up to 3 kGy)	Reduced microbial load but increased lipid oxidation (TBARS). No significant sensory changes.	[87]
Cooking methods (roasting, boiling, sous-vide)	Roasting produced the highest aldehydes (hexanal, 13-fold increase). Sous-vide had lower oxidation but generated sulfur-containing VOCs. Boiling increased furans.	[72]
Tangerine peel (TP) in frying	Reduced carcinogenic HAAs (94% inhibition with 5-year TP). Unique VOCs (d-limonene, thymol) decreased with TP aging.	[88]
Biological factors		
Age at slaughter	Younger rabbits (63 days) had lower intramuscular fat (0.53%) vs. older rabbits (70–80 days; ~1.4–2%).	[83]
Sex differences	Males had higher redness (a*) and shear force (tougher meat) but improved water-holding capacity (WHC) with longer chilling.	[82]
Breed differences	Botucatu rabbits showed different muscle fiber composition vs. hybrids, affecting rigor mortis and tenderness.	[89]
VOC profiles		
VOC diversity	Rabbit meat has fewer VOCs (6) than chicken (29) or beef (28). Profiles stable in fresh meat but diversify during decomposition.	[90]

VOCs = Volatile organic compounds; MUFA = Monounsaturated fatty acids; TBARS = Thiobarbituric acid reactive substances; SFA = Saturated fatty acids; PUFA = Polyunsaturated fatty acids; FSO = Flaxseed oil; ALC = α-Lipoic acid; LCO = Luteolin-coated oil; MDA = Malondialdehyde; WHC = Water-holding capacity; HAAs = Heterocyclic aromatic amines; Se = Selenium; PCA = Principal component analysis; % = percentage; vs. = Versus; kGy = Kilogray; h = Hours; °C = Degrees Celsius; g = Grams; a* = Redness index in colorimetry.

**Table 6:** Summary of key findings on VOCs in eggs and egg quality.

Factor Investigated	Key Findings on VOCs	Key Findings on Egg Quality	Reference
Dietary *Sapindus saponaria* oil (SIO: 0%, 0.5%, 1%)	38 VOCs detected (aldehydes and aromatic hydrocarbons dominant). Flavor compounds varied with SIO levels. PUFAs linked to flavor formation.	Higher sensory scores (nutty, roasted potato) in 0.5% SIO group. Increased PUFAs (ALA, DHA) with SIO. Lower ω-6:ω-3 ratio.	[45]
Management (cage, organic, free-range)	Free-range: 8 VOCs; cage: 15; organic: 11. D-limonene dominant.	Diet/foraging altered aroma/flavor	[92]
Breed (White Leghorn, Hy-line Brown, Jing Fen)	Nonanal, decanal key VOCs. Aldehydes (~80% of profile). Breed influenced VOC distinctions.	—	[42]
Diets (cabbage/onion/rapeseed oil, free-range)	Raw yolks had low VOCs; sulfur compounds increased with rapeseed oil. Free-range eggs had fewer VOCs. Aldehydes formed during cooking.	No impact on shell stiffness/sensory quality. Feed influenced carotenoids, ω-3 fatty acids.	[93]
Embryo sex, fertility, and development	VOCs encode embryo sex/fertility info. Non-invasive detection possible.	—	[94]
Dietary biochar (BC) and biochar-based mixture (BCM)	No significant VOC differences in excreta.	Improved shell resistance (6–10%), egg mass (2–4%). No sensory differences in boiled eggs.	[95]
Raw egg storage time (0–28 days) for salt-baked marinated eggs (SBMEs)	Aldehydes (benzaldehyde, hexanal) dominant. VOC changes faster in yolk than white. Storage time significantly altered profiles.	PUFAs and MUFAs decreased after 28-day storage. Best sensory score at 7 days. Moisture content shifted after 21 days.	[91]
High-voltage cold plasma (HVCP) treatment time (0–300 s)	65 VOCs identified (aldehydes highest). Fluctuating aldehyde concentrations with treatment time.	No change in protein/reducing sugars; mineral content varied.	[96]
Fungal contamination (storage time)	2-Pentanone, 1-Pentanol linked to microbial growth.	Pathogen risk increased with storage.	[97]

VOCs = Volatile organic compounds; SIO = *Sapindus saponaria* oil; PUFAs = Polyunsaturated fatty acids; ALA = Alpha-Linolenic acid; DHA = Docosahexaenoic acid; ω-6:ω-3 = Omega-6 to omega-3 fatty acid ratio; SBMEs = Salt-baked marinated eggs; MUFAs = Monounsaturated fatty acids; HVCP = High-voltage cold plasma; BC = Biochar; BCM = Biochar-based mixture; — = Not applicable or no data reported.

**Table 7:** Characteristic volatile compounds identified in cooked meat of different species (beef, pork, chicken, lamb), their relative contents, and associated odor descriptors based on GC–olfactometry analysis [25].

Species	Class of VOC	VOC	Concentration (µg/g)	Characteristic Odor
Beef	Aldehydes	Hexadecanal	81.41	Cardboard
	Aldehydes	Nonanal	5.39	Fat, citrus
	Aldehydes	Hexanal	2.08	Grass, fat
	Aldehydes	Benzaldehyde	0.12	Almond, burnt sugar
	Alcohols	Z-9-octadecen-1-ol	0.34	Fatty, animal
	Alcohols	1-octen-3-ol	0.16	Mushroom
	Ketones	3-Hydroxy-2-butanone	0.7	Buttery, creamy, fatty, sweet
	Ketones	2-Octadecanone	0.55	Green
	Carboxylic acids	Hexanoic acid	0.89	Sweat
	Carboxylic acids	2,4-Hexadienoic acid	0.21	Acrid
	Esters	Ethyl acetate	50.58	Pineapple
	Esters	Ethyl 9-hexadecenoate	0.18	Fruity
	Furans	5-Methyl-2-acetylfuran	0.71	Nutty
	Furans	Tetrahydrofuran	0.66	Butter, caramel
	Heterocyclic	3,5-Diethyl-1,2,4-trithiocyclopentane	2.85	Beef aroma
Pork	Aldehydes	Nonanal	2.86	Fatty, floral, wax
	Aldehydes	Benzaldehyde	2.53	Bitter almond
	Aldehydes	Octanal	1.97	Fatty, pungent
	Aldehydes	Trans-2-nonenal	1.47	Cucumber, farinaceous, greasy, grassy
	Aldehydes	Heptanal	1.25	Fatty, putty
	Aldehydes	Hexanal	0.95	Green, grass
	Alcohols	3-Methyl-1-butanol	3.1	Pungent
	Alcohols	Hexanol	1.11	Woody, grassy, fruity, metallic
	Alcohols	1-Octen-3-ol	0.83	Mushroom
	Alcohols	3-Methyl-3-buten-1-ol	0.34	Sweet fruity
	Ketones	2-Butanone	0.83	Burnt, chocolate
	Ketones	2-Heptanone	0.8	Citrus, spicy
	Esters	γ-Butyrolactone	0.96	Creamy, sweet
	Esters	Ethyl 2-methylbutanoate	0.35	Fruity, strawberry
	Carboxylic acids	Hexanoic acid	0.81	Goaty
	Carboxylic acids	Nonanoic acid	0.25	Fatty, cheese
	Sulfur compounds	Methional	1.74	Cooked potato, roasted
	Sulfur compounds	Dimethyl disulfide	1.24	Moldy, onion-like
	Pyrazines	2,5-Dimethyl pyrazine	0.24	Nutty, roasted
	Furans	2-Pentylfuran	1.29	Green bean, butter
Chicken	Aldehydes	P-methoxybenzaldehyde	20.9	Anisic, hawthorn-like
	Aldehydes	Benzaldehyde	9.88	Almond, burnt sugar
	Aldehydes	Nonanal	0.73	Fatty, citrus, wax
	Alcohols	1-Octen-3-ol	0.06	Shiitake mushroom
	Ketones	P-methoxypropiophenone	0.39	Musty, anisic
	Esters	Trans vinyl cinnamate	0.92	NR
	Furans	2-Pentylfuran	0.81	Green bean, butter
	Furans	2-Acetylfuran	0.21	Butter, meaty
Lamb	Aldehydes	Hexanal	109.23	Apple, leaf, delicate
	Aldehydes	Heptanal	31.32	Nutty, fruity green
	Aldehydes	(E)-2-nonenal	30.09	Fatty, paper
	Aldehydes	Nonanal	18.25	Fatty, rancid
	Aldehydes	Benzaldehyde	13.09	Almond, burnt sugar
	Alcohols	Hexanol	12.42	Woody, fruity, winey
	Carboxylic acids	4-Methylnonanoic acid	316.73	Sweet muttony
	Carboxylic acids	4-Ethyloctanoic acid	186.22	Sweet muttony
	Carboxylic acids	Acetic acid	5.09	Vinegar
	Esters	Ethyl dodecanoate	6.18	Fatty
	Furans	2-Methyl-5-(methylthio)furan	36.09	Meat, onion
	Furans	2-Pentylfuran	24.21	Green bean, butter
	Pyrazines	2,3,5,6-Tetramethylpyrazine	15.52	Chocolate-like
	Sulfur compounds	Benzyl methyl sulfide	4.88	Roasted, muttony

**Table 8:** Comparative overview of key aromatic fingerprinting techniques used in food volatile analysis, including their detection principles, typical detection and quantification limits, advantages, and limitations.

Technique	Detection Principle	Detection/Quantification Limits	Advantages	Limitations	References
GC–MS	Separation of VOCs on a GC column followed by mass spectral identification	Down to low ppb levels for many volatiles	Gold standard; compound-specific; structural info; quantitative	Time-consuming; costly; requires expertise; limited for highly volatile/reactive compounds	[141,142,143,144,145,146,147]
E-nose	Arrays of semi-selective sensors (MOS, CP, QCM) responding to headspace VOCs	µg/L to mg/L (compound-dependent; not absolute)	Rapid, non-destructive; pattern recognition; spoilage/authenticity detection	No compound-specific info; sensor drift; humidity-sensitive	[149,150,151,152,153,158,159,160]
FTIR spectroscopy	Absorbance of IR radiation by functional groups, generating spectral fingerprints	Typically, ppm range; sensitive to functional group classes	Fast, reagentless, minimal prep; chemometric integration	Overlapping peaks; indirect compound identification; matrix effects	[166,167,168,169,170,171,172]
IMS/GC–IMS	Separation of ionized volatiles by drift time in electric field (±GC pre-sep)	Low ppb detection; quantitative with calibration	High sensitivity; rapid (10–15 min); on-site analysis; 2D fingerprints	Lower resolution than GC–MS; compound identification less robust	[72,105,173,174]
E-tongue	Sensor arrays mimicking taste receptor responses (potentiometric, voltammetric, impedance)	mg/L for salts/organic acids; µM–mM for many tastants	Complementary to E-nose; detects non-volatile taste-active compounds; combined use gives full flavor profile	Less specific than chromatography; cross-sensitivity; needs calibration	[164,175,176,177]

GC = Gas chromatography; MOS = Metal-oxide sensors; CP = Conducting polymers; QCM = Quartz crystal microbalances; FTIR = Fourier Transform Infrared spectroscopy; IR = Infrared; IMS = Ion mobility spectrometry; Ppb = Parts per billion.

## Data Availability

No new data were created or analyzed in this study. Data sharing is not applicable to this article.

## Abbreviations

The following abbreviations are used in this manuscript:

VOCs	Volatile Organic Compounds
HS–GC–MS–O	Headspace–Gas Chromatography–Mass Spectrometry–Olfactometry
GC–MS	Gas Chromatography–Mass Spectrometry
GC–IMS	Gas Chromatography–Ion Mobility Spectrometry
FTIR	Fourier-Transform Infrared Spectroscopy
E-nose	Electronic Nose
OAV	Odor Activity Value
PCA	Principal Component Analysis
PLSR	Partial Least Squares Regression
LDA	Linear Discriminant Analysis
ANN	Artificial Neural Network
QCM	Quartz Crystal Microbalance
MOS	Metal Oxide Semiconductor (sensor)
CP	Conducting Polymer (sensor)
SFA	Saturated Fatty Acids
PUFA	Polyunsaturated Fatty Acids
MUFA	Monounsaturated Fatty Acids
BCFA	Branched-Chain Fatty Acids
IMF	Intramuscular Fat
DM	Dry Matter
CLA	Conjugated Linoleic Acid
ALA	α-Linolenic Acid
EPA	Eicosapentaenoic Acid
DHA	Docosahexaenoic Acid
PDO	Protected Designation of Origin
SIRA	Stable Isotope Ratio Analysis
DNA	Deoxyribonucleic Acid
PCR-RFLP	Polymerase Chain Reaction–Restriction Fragment Length Polymorphism
CRISPR	Clustered Regularly Interspaced Short Palindromic Repeats
TBARS	Thiobarbituric Acid Reactive Substances
MAP	Modified Atmosphere Packaging
VP	Vacuum Packaging
CP (packaging)	Cling-Wrapped Packaging
GP	Genetic Programming
SVM	Support Vector Machine
AFLS	Adaptive Fuzzy Logic System
